# Management of constipation in long-term care hospitals and its ward manager and organization factors

**DOI:** 10.1186/s12912-020-0398-z

**Published:** 2020-01-16

**Authors:** Manami Takaoka, Ayumi Igarashi, Asako Futami, Noriko Yamamoto-Mitani

**Affiliations:** Department of Gerontological Homecare and Long-term Care Nursing, Graduate School of Medicine, the University of Tokyo, 7-3-1 Hongo, Bunkyo-ku, Tokyo, 113-0033 Japan

**Keywords:** Constipation, Assessment, Nursing care, Long-term care, Ward manager

## Abstract

**Background:**

Studies examining organizational factors that may influence constipation management in long-term care (LTC) hospitals are lacking. This study aimed to clarify the practice of constipation management in LTC hospitals and to explore its factors, including ward manager’s perception, organizational climate, and constipation assessment.

**Methods:**

In this cross-sectional questionnaire survey of ward managers and staff nurses working in LTC wards, we determined daily assessment and practices regarding constipation management. We also conducted multivariate analyses to examine factors related to constipation management.

**Results:**

There was a 20% response rate to the questionnaire. Nearly all LTC wards routinely assessed bowel movement frequency; other assessments were infrequent. Laxatives were used, but the use of dietary fiber and probiotic products was implemented in only 20–30% of wards. The implementation of non-pharmacological management and adequate use of stimulant laxatives were positively associated with the ward manager’s belief and knowledge, organizational climate, the existence of nursing records for constipation assessment, planned nursing care for constipation, and organized conferences and in-hospital study sessions on constipation management.

**Conclusion:**

Areas to improve constipation management in LTC hospitals include altering the ward manager’s perception, improving hospital’s organizational climate, and introducing standardized assessment/care planning systems.

## Background

Constipation management is an essential component of long-term care (LTC) for older adults. Constipation can cause discomfort and abdominal pain [1], as well as serious conditions including megacolon, intestinal impaction, or volvulus [2]. It can lower well being [3] and affect healthcare costs [4]. Although the definitions of constipation varies, the prevalence of constipation is high among older adults in LTC settings [5], with a need for daily constipation management [6].

Nurses in LTC hospitals must provide care for older adults with severe physical and cognitive problems [7]. The patient-to-nurse ratio is typically higher in LTC hospitals than in acute care settings. For example, in Japan, government regulations for LTC require this ratio to be 20:1; it is designated to be 7:1 or 10:1 in acute care hospitals [8]. LTC hospital nurses should focus on more technical or acute care procedures that have a direct and immediate impact on patients, as they can only provide minimal basic care due to their busy schedules [9]. It is difficult for LTC hospital nurses to perform additional assessment and management owing to their limited time.

Nursing practice guidelines recommend non-pharmacological management for constipation in addition to appropriate use of laxatives [10]. Non-pharmacological management includes increased fluid intake, increased physical activity, regular encouragement to use the bathroom, and intake of dietary fiber and probiotic products. However, non-pharmacological management is applied infrequently in LTC settings as nurses may be reluctant to change from laxative administration [11]. Furthermore, despite the recommendation of the American Gastroenterological Association that stimulant laxatives should be used sporadically [12], nurses in LTC settings regularly administer them [13]. To promote effective constipation management in LTC hospitals, we should clarify the actual situations and factors associated with constipation management.

Effective constipation management requires bowel movement assessment [10], including consideration of history of laxative use, bowel movement patterns including frequency, stool consistency, and typical bowel movement time, and physical assessment such as palpation of abdominal mass and auscultation of bowel sounds. However, previous studies revealed that bowel movement assessment has been infrequently used according to LTC nursing records [14, 15], and there is little evidence regarding assessment of bowel movements based on nursing practice guideline recommendation. We should clarify how nurses assess and record inpatients’ bowel movements and examine whether assessment could lead to effective constipation management.

Ward managers’ perceptions and the organizational climate in LTC hospitals can contribute to effective constipation management. It has been reported that ward managers who acknowledge the importance of evidence-based nursing practice support their staff in performing evidence-based practices [16, 17]. Additionally, the organizational climate has been related to evidence-based nursing practices in diabetes management [18] or person-centered care [19]. However, little is known about the relationship between ward managers’ perceptions and constipation management. Furthermore, some organizational factors such as case conferences [20], staff resources [21], educational opportunities [17], and nursing care plans [22] can impact nursing practice. These factors should be examined to determine their relationship with constipation management.

Here, we aimed to assess the current constipation management practices in LTC hospitals and to explore the factors related to constipation management, specifically individualized and daily constipation assessment in LTC hospitals, the ward managers’ perception, and the organizational climate.

## Methods

### Definitions

Constipation was defined as the condition in which a person has difficulty in comfortably passing a sufficient amount of stool [23].

### Study design and participants

**Fig. 1:**
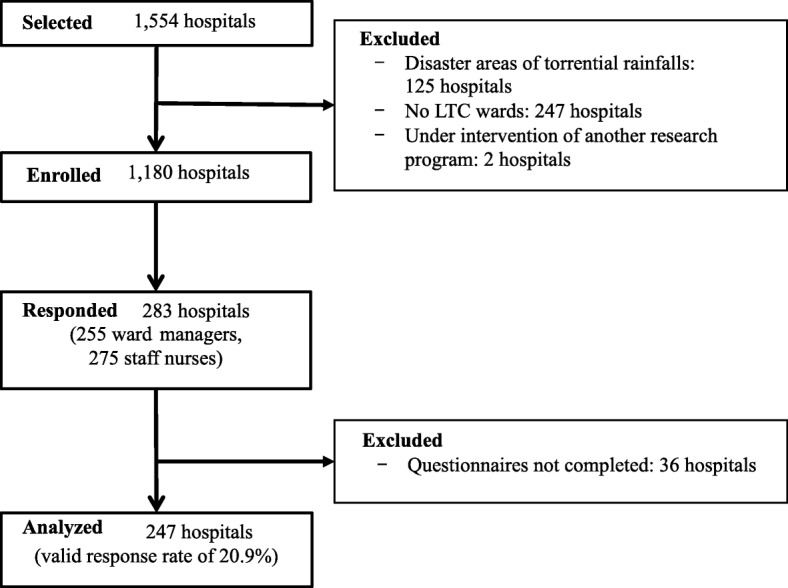
Flow chart of participants in this survey

We conducted a cross-sectional questionnaire survey of ward managers and staff nurses working in LTC wards in Japan from August to September 2018. We randomly selected 1554 hospitals from 3844 hospitals with LTC wards from a hospital database representing all of Japan in 2015 (Fig. 1). Of these, we excluded 125 hospitals in disaster areas associated with torrential rainfalls in Western Japan and 247 hospitals without LTC wards in the Reporting on Medical Functions of Hospital Beds in 2016 [24]. We excluded two hospitals that were under intervention of another research program. Consequently, we extracted 1180 hospitals with LTC wards in this study.

We sent self-administered, anonymous questionnaires to the ward managers and staff nurses of the 1180 hospitals. We then asked the hospital nursing directors to distribute the questionnaires to the LTC ward managers and to one staff nurse within each ward who had a high level of familiarity regarding the state of patients’ bowel management. LTC ward managers and staff nurses who agreed to participate in the survey completed questionnaires and returned these by mail.

In the questionnaire, we explained the purpose and methods of the study, the voluntary nature of participation, and the right to refuse participation. Written informed consent was received from the nurses and ward managers involved in this study. This study was approved by the Research Ethics Committee of the university (No. 12037).

### Measures

In this study, the questions posed to ward managers concerned the general characteristics of hospitals, wards, and inpatients; organizational factors; and ward manager demographic characteristics. The questions asked to staff nurses concerned their demographic characteristics, how constipation management was assessed, and the actual practice of constipation management.

### Characteristics of hospitals, wards, and inpatients

Hospital characteristics included hospital ownership and total number of beds. Ward characteristics included the total number of beds in the ward, average length of patient stay, bed occupancy rate, number of hospitalized patients, number of full-time registered nurses, licensed practical nurses, care workers, and type of reimbursement (types 1 or 2). To be designated as type 1 wards, more than 80% of admitted patients should have high medical acuity levels, whereas in type 2 wards, only more than 50% need to have high acuity levels [9]. We investigated the number of patients using a diaper, those receiving nutrition through a gastric feeding tube, and those receiving total parenteral nutrition to understand the inpatient characteristics for activities of daily living.

### Organizational factors

We inquired about the presence or absence of a certified nurse in wound, ostomy, and continence nursing; education availability for staff nurses (in-hospital and out-hospital study sessions); case conferences; the existence of committees; and nursing care plans for constipated patients.

The organizational climate was reported by staff nurses using a scale [25], rated on a 5-point Likert scale from 1 (never experienced) to 5 (always experience). This instrument comprises the following four subscales: sense of control, staff morale, intimacy, and learning atmosphere. Given that the existing learning opportunity was mostly for facilitating implementation of EBP [17], we only used the learning atmosphere subscale for the analyses. The internal consistency reliability of the learning atmosphere was acceptable (Cronbach’s alpha coefficient = 0.73).

### Ward manager characteristics

The demographics of ward managers included age, sex, and qualification. Work-related variables were measured by years working in current workplace, types of workplace experience, experience in receiving education regarding constipation management (in-hospital study sessions, out-hospital study sessions, books or magazines, and academic conferences), and knowledge on stimulant laxative use. To examine the ward manager’s knowledge on laxative use, we asked whether the stimulant laxatives should be used every day, rated on a 4-point Likert scale ranging from 1 (strongly disagree) to 4 (strongly agree).

We further examined the ward manager’s beliefs and preferences regarding use of laxatives. Regarding beliefs, we asked: “I believe we cannot manage constipation without laxatives.” For preference, we asked: “I want to manage constipation without relying on laxatives.” The answers were rated on a 4-point Likert scale ranging from 1 (strongly disagree) to 4 (strongly agree). To achieve face validity, we asked the nurses in LTC hospitals and the gastroenterologists if all questions were clearly worded and would not be misinterpreted.

### Staff nurse characteristics

The characteristics of staff nurses, including age, sex, qualifications, and years working in their current workplace, were recorded.

### Constipation assessment

Items regarding constipation assessment at the initial intake and on a daily basis were developed based on the nursing practice guidelines [10]. The initial assessment included the following six items: ability to sense the urge to defecate, medical history of laxative use, abdominal mass, bowel sounds, fecal impaction, and hemorrhoids. Daily assessment included the following seven items: frequency of bowel movements, time of bowel movement, amount of stool, abdominal mass, bowel sounds, stool consistency, and use of Bristol Stool Form Scale (BSFS) for assessing stool consistency. We asked the managers whether there was a recording field for each item in the nursing records and whether the nursing staff recorded each item for all, some, or none of the patients.

### Constipation management

Items concerning constipation management were developed based on recommendations from the clinical/nursing practice guidelines for constipation [10, 23, 26]. Daily interventions included the following eight items: osmotic laxatives (magnesium oxide), stimulant laxatives (sodium picosulfate and senna), secretagogues (lubiprostone), Chinese herbal medicine, and medicine for external use and procedures (glycerin enema, suppository laxative, and digital disimpaction). Moreover, we inquired for the daily implementation of the following five non-pharmacological management practices: increased fluid intake, regular encouragement to use bathroom, increased physical activity, using dietary fiber products, and using probiotic products.

We asked the nursing staff to describe the daily constipation management practice in LTC wards based on the abovementioned pharmacological and non-pharmacological managements. In addition, we asked the nursing staff to select three patients with severe constipation and to describe the daily constipation management practice for each patient.

The following outcome variables were used: the total number of non-pharmacological management practices, whether dietary fiber and probiotic products are used in the ward, and non-use of stimulant laxatives on consecutive days. The total number of non-pharmacological management practices reflects effective constipation management with diverse options, because multiple [27] and individually tailored approaches [28] are reported to be effective. The efficacy of dietary fiber and probiotic products has been reported [29, 30]; however, these products are infrequently implemented in the LTC setting [11]. These indicators were measured based on whether they were used daily (1) or not (0). Non-use of stimulant laxatives on consecutive days is recommended by the American Gastroenterological Association [26]. It was measured based on whether staff nurse used the stimulant laxatives on consecutive days for any patient out of the three selected patients (0) or not (1).

### Data analysis

Data analyses were conducted by utilizing data for each ward as a unit of analyses; some data on individual patients were summed together to represent ward characteristics. The variables such as knowledge of laxative use and belief were reversed to high knowledge and belief for constipation management with a high score. First, we generated descriptive statistics. Second, we conducted bivariate analyses to examine associations between constipation management and ward manager’s perceptions, organizational climate, and other variables.

Finally, we conducted multivariate analyses. We used the multiple linear regression analysis for the total number of non-pharmacological management practices and multiple logistic regression analysis for dietary fiber products, probiotic products, and non-use of stimulant laxatives on consecutive days.

The ward manager’s perception and organizational climate of the learning atmosphere were entered in the model using the force-entry method; other variables were entered using the forward selection for variables with *P* < .20. To control for ward patient characteristics (i.e., medical acuity and activities of daily living assistance needs), the number of hospitalized patients per LTC ward, those with a gastric feeding tube, and those receiving total parenteral nutrition were also applied using the force-entry method as independent variables. The significance level was *P* < .05 (two-tailed). All analyses were conducted using SPSS Statistics for Windows, version 25.0. (Armonk, NY: IBM Corp).

## Results

Among the 1180 hospitals, 283 returned questionnaires. Among these, 36 hospitals did not return the completed questionnaires for both ward managers and nursing staff; therefore, we only analyzed data from 247 hospitals (valid response rate, 20.9%).

### Hospital, ward, and participant characteristics

Bed occupancy rates in this study were 88.7% ± 13.8%, comparable with the results of our previous study [31] on LTC wards (89.2% ± 13.5%). The average length of stay was 324 ± 329 days, which was longer than that reported in our previous study (240.2 ± 144 days) [31]. More than 70% of the LTC wards participating in this study were reimbursed using the type 1 reimbursement system, indicating that they had a higher number of inpatients with high medical acuity levels, such as patients with intractable disease and patients using ventilator. Among the 42.0 ± 11.7 inpatients in the ward, the number of patients using a diaper was 34.9 ± 13.4 (83% of the total inpatients) (Table 1).

**Table 1:** Characteristic of hospital/wards, ward manager, and staff nurses (*n* = 247)

	n (%)	
	Mean ± SD	Range
Characteristic of hospital and wards		
Hospital ownership		
Public interest corporations	26 (10.7)	
Social welfare corporations	2 (0.8)	
Medical corporations	195 (80.6)	
Others	19 (7.9)	
Total number of hospital beds	166.3 ± 106.7	〔31–920〕
Total number of beds in the ward	47.4 ± 10.6	〔9–94〕
Average length of stay	323.5 ± 329.3	〔18–1837〕
Bed occupancy rate	88.7 ± 13.8	〔15–100〕
Number of hospitalized patients per ward	42.0 ± 11.7	〔7–90〕
Full-time staff rate		
RNs/RNs + LPNs	0.7 ± 0.2	〔0.2–1〕
RNs + LPNs+CW/total number of beds in the ward	0.5 ± 0.1	〔0.1–0.9〕
RNs + LPNs/RNs + LPNs+CW	0.3 ± 0.9	〔0.1–0.6〕
Charged hospitalization basic rate		
Type 1 (> 80% high medical acuity patient)	166 (73.8)	
Type 2 (> 50% high medical acuity patient)	49 (21.8)	
Interim measure 1	10 (4.4)	
Number of patients using a diaper	34.9 ± 13.4	〔0–62〕
Number of patients receiving nutrition through a GFT	16.8 ± 10.8	〔0–58〕
Number of patients receiving TPN	6.9 ± 8.7	〔0–48〕
Organizational system of CM		
Certified Nurse in WOCN	26 (10.6)	
Staff participation in in-hospital study sessions	82 (10.7)	
Staff participation in out-hospital study sessions	57 (33.5)	
Case conference for CM in unit	124 (23.4)	
Committee or group that focus on CM	22 (8.9)	
Creating nursing care plans for CM	128 (54.5)	
Organizational climate of learning atmosphere	9.1 ± 2.2	〔4–15〕
Characteristics of the ward managers		
Age	51.4 ± 7.8	〔28–67〕
Sex (female)	236 (95.5)	
Qualifications (RN)	244 (98.8)	
Years working in the current hospital	17.1 ± 9.8	〔1–49〕
Years working in the current wards	6.0 ± 5.7	〔0–35〕
Years working as a current ward manager	4.0 ± 3.9	〔0–18〕
Past workplace experience		
Visiting nurse	28 (11.7)	
Nurse in a general or university hospital	145 (60.7)	
Long-term care health facility nurse	23 (9.7)	
Educational opportunities of CM		
In-hospital study session	124 (52.3)	
Out-hospital study session	85 (35.9)	
Book or magazine	149 (62.9)	
An academic conference	24 (10.2)	
Knowledge of stimulant laxatives	3.1 ± 0.6	〔2–4〕
Perception of CM		
Beliefs regarding use of laxatives	2.3 ± 0.7	〔1–4〕
Preference of using laxatives	3.1 ± 0.7	〔1–4〕
Staff nurse characteristics		
Age	45.6 ± 9.2	〔22–65〕
Sex (female)	233 (94.3)	
Qualifications (RN)	211 (85.4)	
Years working in the current hospital	13.0 ± 9.4	〔0.5–45〕
Years working in the current wards	5.2 ± 5.1	〔0–30〕

Note: Missing data were excluded from this analysis and percentages for each item were calculated after excluding missing values. *Abbreviations*: *SD* standard deviation, *RN* registered nurse, *LPN* licensed practical nurse, *CW* care worker, *GFT* gastric feeding tube, *TPN* total parenteral nutrition, *CM* constipation management, *WOCN* wound, ostomy and continence nursing

### Constipation assessment and management

At the initial intake, on the day of admission, in more than half of the wards (69.9%), nurses had fields for recording a medical history of laxative use, and in 63.9% of the wards, nurses recorded laxative use as a part of the medical history (Table 2). For daily assessments, in almost all wards, nurses recorded the frequency of bowel movements. Nurses recorded stool consistency in 119 (48.8%) wards but only 58 (23.5%) used the BSFS.

**Table 2:** Hospitalization first-day and daily assessment for older adults with constipation (*n* = 247)

	Record fields		Recorded					
	Presence		All patients		Some patients		Not recorded	
	n (%)		n (%)		n (%)		n (%)	
First day of admission assessment								
The ability to sense the urge to defecate	110	(46.8)	101	(41.4)	68	(27.9)	75	(30.7)
Medical history of laxative use	167	(69.9)	156	(63.9)	62	(25.4)	26	(10.7)
Abdominal mass	64	(26.9)	38	(15.6)	143	(58.6)	63	(25.8)
Bowel sounds	60	(25.2)	39	(15.9)	140	(57.1)	66	(26.9)
Fecal impaction	19	(8.0)	7	(2.9)	49	(20.0)	189	(77.1)
Hemorrhoids	28	(11.7)	12	(4.9)	83	(33.9)	150	(61.2)
Daily assessment								
Frequency of bowel movements	243	(99.2)	244	(98.8)	2	(0.8)	1	(0.4)
Time of bowel movement	77	(32.6)	62	(26.2)	62	(26.2)	113	(47.7)
Amount of stool	165	(67.9)	166	(67.8)	63	(25.7)	16	(6.5)
Abdominal mass	83	(34.7)	25	(10.2)	188	(76.7)	32	(13.1)
Bowel sounds	88	(36.7)	25	(10.2)	192	(78.0)	29	(11.8)
Stool consistency	147	(61.5)	119	(48.8)	104	(42.6)	21	(8.6)
Stool consistency (using the BSFS)	59	(24.9)	58	(23.5)	12	(4.9)	177	(71.7)

Note: Missing data were excluded from this analysis and percentages for each item were calculated after excluding missing values. *Abbreviations*: *BSFS* Bristol Stool Form Scale

In almost all wards, nurses used magnesium oxide (97.5%), sodium picosulfate (95.5%), and senna (89.3%) (Table 3). Notably, non-pharmacological management practices varied; more than half of the nurses provided regular encouragement to increase patients’ fluid intake and bathroom use (55.0 and 52.1%, respectively); in contrast, physical activity, dietary fiber products, and probiotic products were facilitated or encouraged in 22.3, 34.4, and 20.7% of wards, respectively. Consecutive use of stimulant laxatives was reported in 70% of the wards.

**Table 3:** Daily management for older adults with constipation (*n* = 247)

		n (%)	
		Mean ± SD	
Osmotic laxatives	Magnesium oxide	238	(97.5)
Stimulant laxatives	Sodium picosulfate	233	(95.5)
	Senna	217	(89.3)
Secretagogues	Lubiprostone	46	(19.5)
Chinese herbal medicine		119	(51.1)
External use medicine or procedure	Glycerin enema	158	(64.2)
	Suppository laxative	174	(71.9)
	Digital disimpaction	208	(85.6)
Non-pharmacological management	Increased fluid intake	132	(55.0)
	Regular encouragement to use bathroom	126	(52.1)
	Increased physical activity	53	(22.3)
	Dietary fiber product	83	(34.4)
	Probiotic product	50	(20.7)
The total number of non-pharmacological managements		1.8	1.4

Note: Missing data were excluded from this analysis and percentages for each item were calculated after excluding missing values

### Factors related to daily practices of constipation management

We examined the factors of daily constipation management using multiple regression analyses. The total number of non-pharmacological management practices was positively associated with the creation of nursing care plans for constipation management (β = 0.19; *P* = .008), the organizational climate of the learning atmosphere (β = 0.13; *P* = .069), and the belief of ward managers regarding use of laxatives (β = 0.14; *P* = .051) (Table 4).

**Table 4:** Association between daily non-pharmacological management practice and ward manager’s perception and organizational characteristics

	The number of NPMs								
	*n* = 180								
		95%CI							
	*β*	*LL–UL*	*p*						
Characteristics of the organization									
Creating nursing care plan for CM (ref: non-existent)	0.19	0.14–0.92	0.008						
Organizational climate of the learning atmosphere	0.13	−0.01–0.17	0.069						
Characteristics of ward manager									
Perception; beliefs regarding use of laxatives	0.14	−0.001–0.57	0.051						
Perception; preference of using laxatives	0.06	−0.17–0.41	0.407						
Adjusted R^2^			0.167						
	Dietary fiber product			Probiotic product			Non-use of SL on the consecutive day		
	*n* = 202			*n* = 217			*n* = 181		
		95%CI			95%CI			95%CI	
	*OR*	*LL–UL*	*p*	*OR*	*LL–UL*	*p*	*OR*	*LL–UL*	*p*
Characteristics of the organization									
Case conference for BM in the ward (ref: non-existent)	2.26	1.12–4.28	0.012						
Organizational climate of the learning atmosphere	0.98	0.85–1.13	0.795	1.30	1.11–1.53	0.001	1.09	0.93–1.28	0.281
RF of time of bowel movement (ref: non-existent)	2.69	1.40–5.18	0.003						
RF of stool consistency using BSFS (ref: non-existent)				2.81	1.31–6.06	0.008	2.77	1.28–5.99	0.01
Characteristics of ward manager									
Age							0.95	0.90–0.99	0.014
Participation in the in-hospital study session	2.31	1.21–4.43	0.012						
Knowledge of stimulant laxative							2.34	1.23–4.46	0.01
Perception; beliefs regarding use of laxatives	1.31	0.83–2.06	0.251	1.69	1.04–2.75	0.035	0.71	0.42–1.20	0.201
Perception; preference of using laxatives	1.38	0.87–2.21	0.175	0.72	0.44–1.17	0.185	0.70	0.41–1.18	0.18
Nagelkerke R^2^	0.181			0.146			0.171		

Note: Missing data were excluded from this analysis and percentages for each item were calculated after excluding missing values. *Abbreviations*: *NPM* non-pharmacological management, *CM* constipation management, *BM* bowel management, *RF* record fields, *BSFS* Bristol Stool Form Scale, *CI* confidence interval, *OR* odds ratio, *LL* lower limit, *UL* upper limit, *ref*. reference

The following variables were controlled: the number of hospitalized patients per LTC ward, the number of patients receiving nutrition by gastric feeding tube, and the number of patients receiving total parenteral nutrition. The following variables were used via the forced entry method: organizational climate of the learning atmosphere, beliefs regarding use of laxatives, Preference of using laxatives

The following variables were used besides the independent variables used in forward selection to assess each outcome: The number of NPM; the average length of stay, bed occupancy rate, the number of patients using a diaper, the availability of case conferences regarding CM in the wards, RF of amount of stool. Use of dietary fiber products; the number of patients using a diaper, staff participation in in-hospital study sessions, staff participation in out-hospital study sessions. Use of probiotic products; ward manager’s participation in out-hospital study sessions, staff participation in out-hospital study sessions. Non-use of SL on the consecutive day; the average length of stay, RF of amount of stool, age of ward manager, ward manager’s participation in an academic conference, staff participation in out-hospital study sessions

Use of dietary fiber products was positively associated with case conferences for discussing constipation management (odds ratio (OR) = 2.26; *P* = .012), existing record fields for assessing bowel movement time (OR = 2.69; *P* = .003), and participation of the ward managers in in-hospital study sessions for constipation management (OR = 2.31; *P* = .012).

Use of probiotic products was positively associated with the organizational climate of learning atmosphere (OR = 1.30; *P* = .001), existing record fields for assessing stool consistency using BSFS (OR = 2.81; *P* = .008), and beliefs of ward managers regarding use of laxatives (OR = 1.69; *P* = .035). Non-use of stimulant laxatives on consecutive days was positively associated with the existing record fields of stool consistency using BSFS (OR = 2.77; *P* = .01) and ward manager’s knowledge of adequate use of stimulant laxative (OR = 2.34; *P* = .01), but it was negatively associated with ward manager’s age (OR = 0.95; *P* = .014).

## Discussion

In this study, we investigated the constipation management and assessment practices in LTC wards in Japan. Practicing constipation management was associated with organizational factors and ward manager. To the best of our knowledge, this is the first study to describe the practice of constipation management/assessment in LTC wards.

In almost all LTC wards, nurses assessed the daily frequency of bowel movements, but other characteristics of elimination (e.g., stool consistency and amount) were not as extensively examined. Given that nurses determined the presence of constipation based on bowel movement frequency in many cases [32], it is habitually recorded in LTC hospitals. Conversely, the implementation of other assessments varied from 10 to 68% despite the nursing guideline recommendation of the importance of multiple assessments [10]. Although 50% of ward nurses assessed stool consistency, only 20% used BSFS, suggesting a lack of assessment with standardized tools in LTC wards. Adding a recording field for assessment with a standardized tool, such as BSFS, could facilitate constipation assessment and result recording.

In almost all LTC wards, nurses used laxatives daily, which was consistent with previous studies [7, 33]. Contrary to the recommendation of clinical guidelines [12, 23], 70% of ward nurses used stimulant laxatives on consecutive days, indicating that healthcare providers in LTC settings do not use them properly.

LTC ward nurses did not implement non-pharmacological management as frequently as the use of laxatives. They reported increased fluid intake and regular encouragement to use the bathroom. Meanwhile, only 20–30% of ward nurses implemented the intake of dietary fiber and probiotic products, a relatively new but costly approach, which has been reported in a previous study [11]. Given that LTC wards in Japan are financed by the pay per capita system, LTC ward staff may face financial challenges regarding the introduction of such a novel approach for constipation management.

Certain characteristics of LTC ward managers, including their perception of constipation management and their knowledge of stimulant laxative use, were associated with practicing constipation management. Ward manager support has been shown to enhance nurses’ evidence-based practice [16]. Therefore, to facilitate effective constipation management, LTC ward managers should have the knowledge of and perceive the importance of constipation management.

The existence of nursing record fields for constipation assessment, including the standardized assessment tool, and a nursing care plan for constipation were also associated with the practice of constipation management. The result is consistent with that of a previous study reporting that care planning for laxative use was related to its actual use [33]. Thus, modifying the organizational recording system to include a standardized assessment tool, such as BSFS, and standardized care planning for constipation management could promote effective constipation management.

The conference and organizational climate were associated with the use of non-pharmacological management and implementation of probiotic and dietary fiber products, rather than laxative use. The results suggest that while the use of medication could be changed only by intervention from the ward managers and the recording system, non-pharmacological constipation management, which is time-consuming and costly, would need an additional approach to promote discussion and care in the organization.

Based on the study results, we recommend two major strategies targeted at ward managers and hospital organization to improve constipation management in LTC hospitals. For LTC ward managers, interventions to facilitate and support successful constipation management [34] might be useful to change their beliefs on use of laxatives. As for the hospital organization, changing the recording system (e.g., integrating evidence-based assessment and management into nursing records), conducting audits and regularly providing feedback during case conferences [35], and increasing learning opportunities (e.g., organizing training sessions in hospitals) may contribute to the promotion of effective constipation management by nurses.

This study has several limitations. First, the cross-sectional design prevented us from concluding a causal relationship among variables. Second, we only asked one staff member about the organizational climate; therefore, it may not have been adequately assessed. Third, the low response rate in this study may lead to sampling bias; nonetheless, we attempted to increase the response rate by limiting the number of participants in each hospital. As the responders tended to be interested in constipation care compared with the non-responders, the practice of non-pharmacological management may be overestimated. Finally, we could not determine the constipation management for each individual patient. Patients’ physical conditions and causes of constipation vary. In future studies, we should clarify the constipation management for each patient to determine effective strategies according to their individual condition.

## Conclusions

Our data revealed that the assessment and practice of constipation management were not conducted sufficiently. Constipation management implementation was associated with both ward manager and organizational factors, including ward manager’s perception and knowledge of laxative use, organizational climate, care planning, assessment, and conferences. Interventions by ward managers and organization would promote more effective constipation management in LTC hospitals.

## Data Availability

The datasets used and/or analyzed during the current study are available from the corresponding author on reasonable request.

## Abbreviations

BSFS: Bristol stool form scale
EBP: Evidence-based practice
LTC: Long-term care
